# Biliverdin Reductase A (BVRA) Mediates Macrophage Expression of Interleukin-10 in Injured Kidney

**DOI:** 10.3390/ijms160922621

**Published:** 2015-09-18

**Authors:** Zhizhi Hu, Guangchang Pei, Pengge Wang, Juan Yang, Fengmin Zhu, Yujiao Guo, Meng Wang, Ying Yao, Rui Zeng, Wenhui Liao, Gang Xu

**Affiliations:** 1 Division of Nephrology, Tongji Hospital, Tongji Medical College, Huazhong University of Science and Technology, 1095 Jiefang Ave, Wuhan 430030, Hubei, China; E-Mails: christina_418@126.com (Z.H.); pgc2008@sina.com (G.P.); 15939991300@163.com (P.W.); amy19861203@126.com (J.Y.); zhufm1988@163.com (F.Z.); 18702765105@163.com (Y.G.); nishuihan2003@163.com (M.W.); yaoyingkk@126.com (Y.Y.); xugang@tjh.tjmu.edu.cn (G.X.); 2 Department of Geriatrics, Tongji Hospital, Tongji Medical College, Huazhong University of Science and Technology, 1095 Jiefang Ave, Wuhan 430030, Hubei, China

**Keywords:** macrophages, biliverdin reductase, interleukin-10

## Abstract

Biliverdin reductase A is an enzyme, with serine/threonine/tyrosine kinase activation, converting biliverdin (BV) to bilirubin (BR) in heme degradation pathway. It has been reported to have anti-inflammatory and antioxidant effect in monocytes and human glioblastoma. However, the function of BVRA in polarized macrophage was unknown. This study aimed to investigate the effect of BVRA on macrophage activation and polarization in injured renal microenvironment. Classically activated macrophages (M1macrophages) and alternative activation of macrophages (M2 macrophages) polarization of murine bone marrow derived macrophage was induced by GM-CSF and M-CSF. M1 polarization was associated with a significant down-regulation of BVRA and Interleukin-10 (IL-10), and increased secretion of TNF-α. We also found IL-10 expression was increased in BVRA over-expressed macrophages, while it decreased in BVRA knockdown macrophages. In contrast, BVRA over-expressed or knockdown macrophages had no effect on TNF-α expression level, indicating BVRA mediated IL-10 expression in macrophages. Furthermore, we observed in macrophages infected with recombinant adenoviruses BVRA gene, which BVRA over-expressed enhanced both INOS and ARG-1 mRNA expression, resulting in a specific macrophage phenotype. Through *in vivo* study, we found BVRA positive macrophages largely existed in mice renal ischemia perfusion injury. With the treatment of the regular cytokines GM-CSF, M-CSF or LPS, excreted in the injured renal microenvironment, IL-10 secretion was significantly increased in BVRA over-expressed macrophages. In conclusion, the BVRA positive macrophage is a source of anti-inflammatory cytokine IL-10 in injured kidney, which may provide a potential target for treatment of kidney disease.

## 1. Introduction

Macrophages belong to the mononuclear phagocytic system and its phenotype controls the homeostasis, immune responses and renal outcomes in normal and injured kidneys [1,2]. Plasticity and functional polarization are features of the mononuclear phagocyte system [3]. In response to the environmental stimuli, the macrophages are divided into several populations with different functions as classically activated macrophages (M1) and alternatively activated macrophages (M2) [4]. M1 macrophages represent the capacities to initiate inflammatory response, carry out anti-microbial function; M2 macrophages promote wound healing, antagonize destructive inflammation, and suppress adaptive immunity [5,6]. M1 phenotype macrophages induce type I helper T cell (Th1) response, typically activated by bacterial moieties (LPS) and Th1 cytokine interferon-gamma (IFN-γ), TNF-α or GM-CSF [7,8,9]. In contrast, M2 phenotype macrophages are involved in type II helper cell (Th2) response, activated by IL-4, IL-13, IL-10, M-CSF or TGF-β [7,8,9]. Macrophages always accommodate their phenotype and function in response to a variety of environments. These polarized processes are generally controlled by some key signaling molecules or genes, such as ARF [10], or TREM-1[11].

Biliverdin reductase A (BVRA) is a well-characterized signaling molecule with serine/threonine/tyrosine kinase activation [12], and it also functions as the only metabolic enzyme that converts biliverdin to bilirubin, which has been reported as a key regulator in the cellular redox cycle [13,14]. Wegiel *et al.* reported that BVRA regulates TLR4 expression and secretion of pro-inflammatory cytokines in monocytes, while BVRA depletion results in increased TLR4 and TNF-α [15], suggesting BVRA is a potential regulator of inflammation and mononuclear phagocyte function. However, Ferenbach *et al.* demonstrated that up-regulation of hemeoxygenase-1(HO-1), the upstream enzyme of BVRA, induced anti-inflammatory macrophage phenotype with increased IL-10 [16,17]. These previously studies indicated that the HO-1/BVRA pathway plays a critical role in the regulation of the inflammatory cytokines release, while the effect of HO-1/BVRA system on macrophage activation and polarization still needs to be specifically investigated.

This study was aimed to detect the BVRA expression in macrophages’ polarization, evaluate the influence of BVRA on IL-10 and TNF-α production in bone marrow derived polarized macrophages and assess the potential therapeutic effect of over-expressed BVRA macrophages on renal injury and repair.

## 2. Results

### 2.1. M1 and M2 Macrophage Polarization of Bone Marrow Derived Macrophage was Induced by GM-CSF and M-CSF 

**Figure 1:**
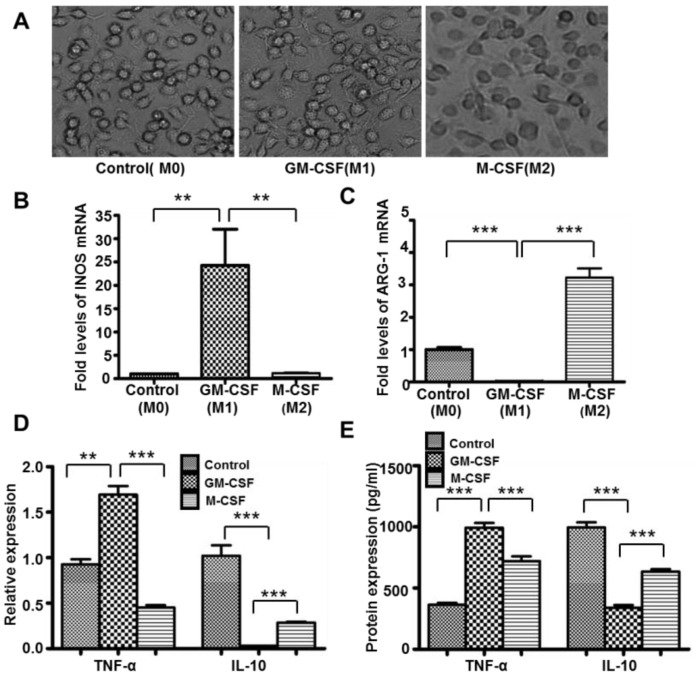
Macrophage polarization was induced successfully *in vitro*. Macrophages’ morphology was observed by light microscope, (M0) control macrophages (untreated macrophages); (M1) macrophages stimulated with GM-CSF; (M2) macrophages stimulated with M-CSF (**A**). Original magnification ×400. INOS (**B**), ARG-1(**C**), IL-10 and TNF-α (**D**) mRNA expression were detected by qRT-PCR. IL-10 and TNF-α (**E**) protein expression were detected by ELISA. *n* = 4, ** *p* < 0.01, *** *p* < 0.001. M0: Macrophages with no treatment; M1: Macrophages treated with GM-CSF; M2: Macrophages treated with M-CSF.

Morphology of adherent macrophages was assessed after seven days of culture with L929 supernatant. The untreated macrophages (M0) exhibited a mixture of round and spindle shaped cells; macrophages treated with GM-CSF (M1) showed a predominantly spindle-like shaped; macrophages treated with M-CSF (M2) changed their morphology into a predominantly circular shape (Figure 1A). Macrophages stimulated with GM-CSF showed elevated mRNA expression of M1 marker INOS (Figure 1B), while M-CSF treated macrophages showed increased M2 marker ARG-1 mRNA expression (Figure 1C). Meanwhile, GM-CSF treated macrophages were associated with a significant down-regulation of anti-inflammatory cytokine IL-10, and increased secretion of pro-inflammatory cytokine TNF-α (Figure 1D,E). These data suggests that macrophage polarization was successfully induced by GM-CSF and M-CSF *in vitro*. We also successfully induced macrophage polarization by LPS and IFN-γ, IL-4 and IL-13, detected INOS and ARG-1 by Western blotting (Supplementary Figure S1A), TNF-α and MMR (CD206) by qRT-PCR (Supplementary Figure S1B,C).

### 2.2. BVRA Expression Increased in M2 macrophage, Compared to M1 macrophage

**Figure 2:**
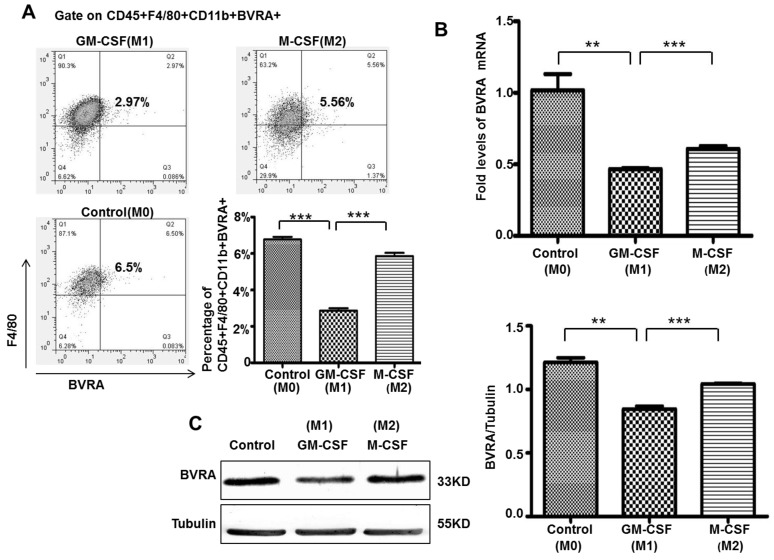
BVRA expression increased in M2 macrophage, compared to M1 macrophage. Macrophages cultured for seven days and further stimulated with GM-CSF and M-CSF for 24 h. BVRA positive macrophages (CD45+F4/80+CD11b+BVRA+) were analyzed by FACS (**A**); BVRA mRNA expression were analyzed by qRT-PCR (**B**); BVRA protein expression were analyzed by Western blotting (**C**). *n* = 4, *** *p* < 0.001, ** *p* < 0.01. M0: Macrophages with no treatment; M1: Macrophages treated with GM-CSF; M2: Macrophages treated with M-CSF.

Using flow cytometry (Figure 2A), we detected an increase BVRA co-expression in M2 macrophages compared to M1 macrophages (5.56% *vs*. 2.97%). Furthermore, we detected the BVRA mRNA expression by qRT-PCR. BVRA mRNA expression increased in M2 macrophage, compared to M1 macrophage (Figure 2B). BVRA protein expression increased in M2 macrophage, compared to M1 macrophage (Figure 2C). But the BVRA expression was decreased in both GM-CSF and M-CSF treated macrophages compared to the control group (Figure 2A–C).

### 2.3. BVRA Mediated IL-10 Expression in Macrophages

**Figure 3:**
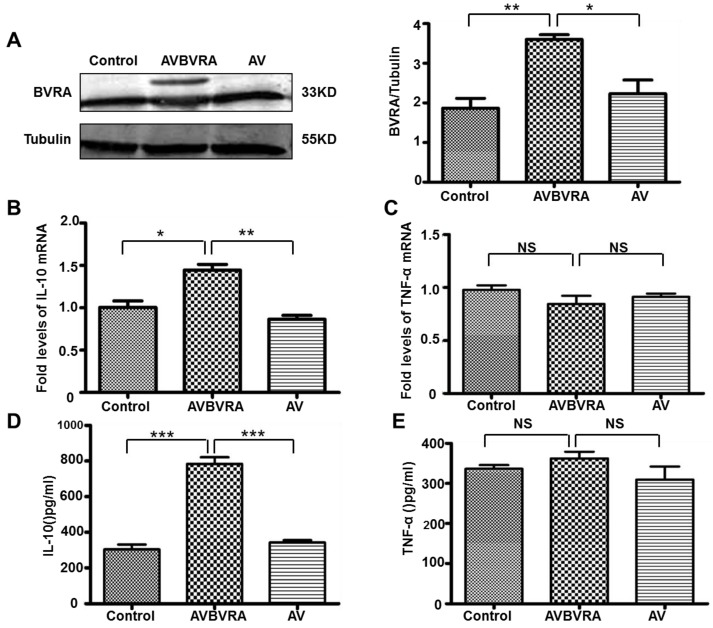
BVRA over-expressed macrophages showed increased IL-10 expression. Macrophages cultured for seven days and transfected with AV-BVRA for 48 h, Western blotting was used to detect the transfection efficiency (**A**) (*p* < 0.05), IL-10 (**B**) and TNF-α (**C**) mRNA expression were detected by qRT-PCR; IL-10 (**D**) and TNF-α (**E**) protein expression were detected by ELISA. *n* = 4, *** *p* < 0.001, ** *p* < 0.01, * *p* < 0.05. NS: no statistical significance. Control: Macrophages with no virus; AVBVRA: Macrophages transfected with AV-BVRA; AV: Macrophages transfected with AV-negative control.

**Figure 4:**
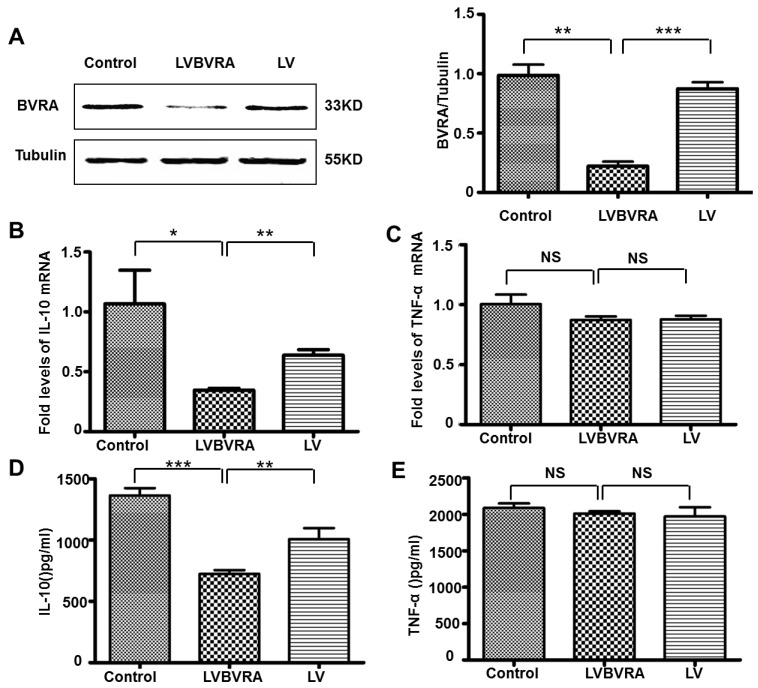
BVRA knockdown macrophages showed decreased IL-10 expression. Macrophages cultured for seven days and transfected with LV-BVRA for 72 h, Western blotting was used to detect the efficiency of knockdown (**A**) (*p* < 0.001), IL-10 (**B**) and TNF-α (**C**) mRNA expression were detected by qRT-PCR; IL-10 (**D**) and TNF-α (**E**) protein expression were detected by ELISA. *n* = 4, *** *p* < 0.001, ** *p* <0.01, * *p* < 0.05. NS: no statistical significance. Control: Macrophages with no virus; LVBVRA: Macrophages transfected with LV-BVRA; LV: Macrophages transfected with LV-negative control.

To determine if BVRA directly regulates pro-inflammatory or anti-inflammatory cytokine production, we used recombinant adenoviruses *in vitro* to deliver BVRA gene into macrophages. BMDM cells were cultured for seven days, and transfected with AV-BVRA virus for 48 h. BVRA protein expression (Figure 3A) and IL-10 expression (Figure 3B,D) were significantly increased in AV-BVRA macrophages, whereas TNF-α expression (Figure 3C,E) was not different between the groups. In Figure 3A, BVRA was detected as the double bands in over-expressed BVRA macrophages as the AVBVRA virus had the GFP expression. The upper band is the transfected BVRA expression fused with GFP, and the low band is endogenous BVRA expression. In BVRA knockdown (LV-BVRA) BMDM cells, IL-10 expression was decreased 50% in (Figure 4B,D) compared to the control LV macrophages. In contrast, TNF-α expression (Figure 4C,E) showed no difference between the groups. 

**Figure 5:**
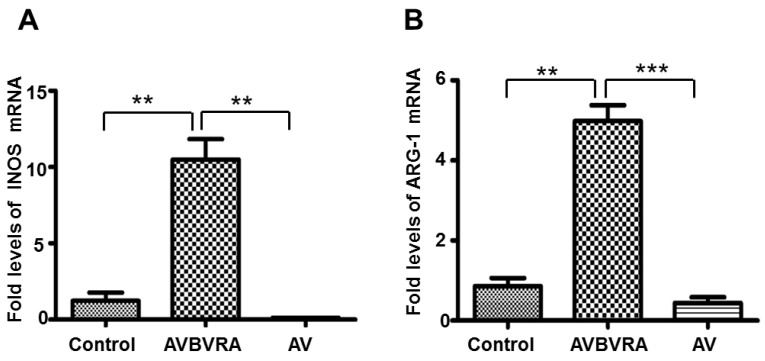
BVRA over-expressed macrophages showed increased INOS and ARG-1 expression levels. INOS (**A**) and ARG-1 (**B**) mRNA expression were detected by qRT-PCR in over-expressed BVRA macrophages; *n* = 4, *** *p* < 0.001, ** *p* < 0.01. Control: Macrophages with no virus; AVBVRA: Macrophages transfected with AV-BVRA; AV: Macrophages transfected with AV-negative control.

Furthermore, we detected the effect of BVRA on macrophage polarization. INOS (Figure 5A) mRNA expression was increased 10-fold in BVRA over-expressed macrophages compared to the control group. ARG-1(Figure 5B) mRNA expression was increased five-fold in BVRA over-expressed macrophages. It suggests that BVRA does not shift macrophage polarization. Taken together, these data indicate that BVRA over-expressed results in a specific macrophage phenotype which is characterized as enhanced anti-inflammatory cytokine IL-10 secretion. Actually, we also had detected several signal pathways molecules such as P-AKT, T-AKT, P-ERK, T-ERK, P-JNK and T-JNK, and found that there was no difference between the AVBVRA group and control group in these signal molecules (Supplementary Figure S2).

### 2.4. BVRA Positive Macrophages Existed in Vivo Renal Tissue.

**Figure 6:**
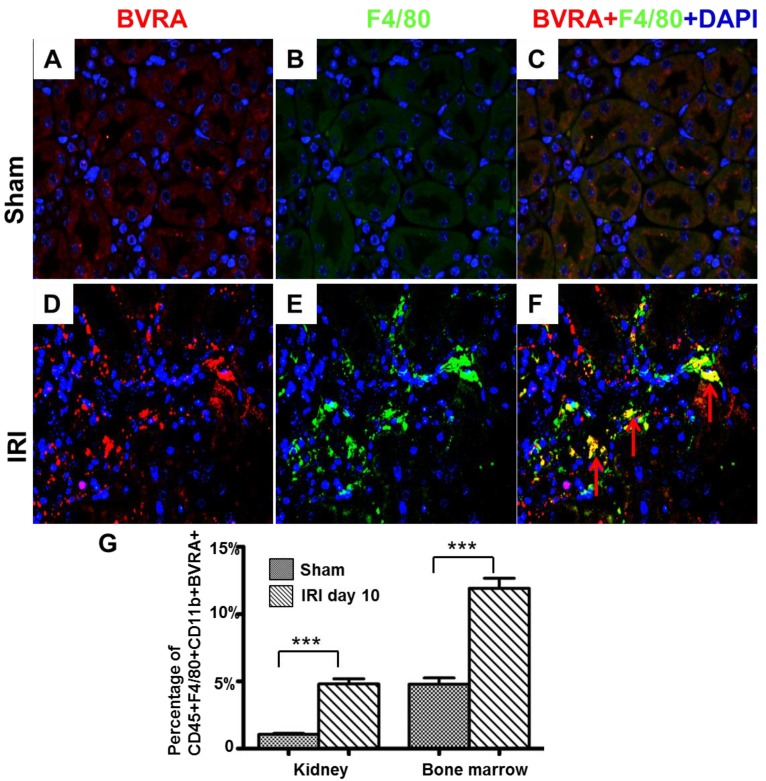
BVRA positive macrophages existed in mice renal ischemia reperfusion injury. Paraffin embedded sham mice renal tissue showed BVRA staining in red (**A**), F4/80 staining of macrophages in green (**B**) and DAPI counterstain in blue. BVRA positive macrophages were merged in yellow (**C**); Paraffin embedded mice ischemia reperfusion injury renal tissue showed BVRA staining in red (**D**); F4/80 staining of macrophages in green (**E**) and DAPI counterstain in blue. BVRA positive macrophages were merged in yellow (**F**) (red arrow). Original magnification 800×; (**G**) Representative CD45+F4/80+CD11b+BVRA cells expression by FACS in the kidney and bone marrow of the sham group and the group 10 days after renal ischemia reperfusion injury. *n* = 4, *** *p* < 0.001.

BVRA positive macrophages were detected by immunofluorescence in mice renal ischemia reperfusion injury (IRI). BVRA in red (Figure 6A,D), F4/80 was stained in green (Figure 6B,E), F4/80 and BVRA merged in yellow (Figure 6C,F). BVRA and F4/80 positive is little in sham renal interstitium but BVRA and F4/80 double positive macrophages were largely scattered in mice IRI renal interstitium. Those results verify that BVRA positive macrophages exist in mice renal ischemia reperfusion injury. In mice renal ischemia reperfusion injury (IRI), BVRA positive macrophages were also detected by FACS in kidney and bone marrow. CD45+F4/80+CD11b+BVRA+ cells were increased in kidney and bone marrow in the group of day 10 after renal ischemia perfusion injury compared to the sham group (Figure 6G).

### 2.5. GM-CSF, M-CSF or LPS Increased IL-10 Expression in BVRA Positive Macrophages

**Figure 7:**
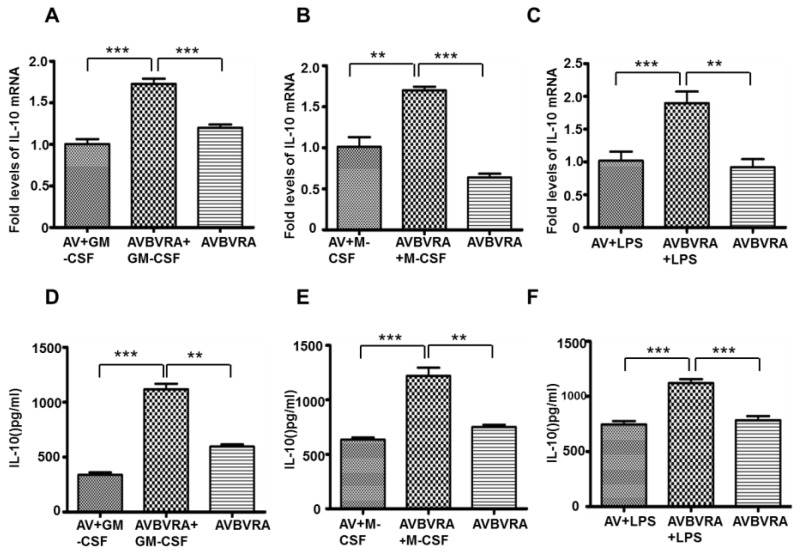
GM-CSF, M-CSF, or LPS increased IL-10 expression in BVRA positive macrophages. (**A**) IL-10 mRNA expression were detected by qRT-PCR in AV-BVRA macrophages stimulated with GM-CSF; (**B**) IL-10 mRNA expression were detected by qRT-PCR in AV-BVRA macrophages stimulated with M-CSF; (**C**) IL-10 mRNA expression were detected by qRT-PCR in AV-BVRA macrophages stimulated with LPS; (**D**) IL-10 protein expression were detected by ELISA in AV-BVRA macrophages stimulated with GM-CSF; (**E**) IL-10 protein expression were detected by ELISA in AV-BVRA macrophages stimulated with M-CSF; (**F**) IL-10 protein expression were detected by ELISA in AV-BVRA macrophages stimulated with LPS. *n* = 4, *** *p* < 0.001, ** *p* < 0.01. AVBVRA: macrophages transfected with AV-BVRA; AV+GM-CSF: macrophages transfected with AV-negative control virus and stimulated with GM-CSF; AVBVRA+GM-CSF: Macrophages transfected with AV-BVRA virus and stimulated with GM-CSF; AV+M-CSF: Macrophages transfected with AV-negative control virus and stimulated with M-CSF; AVBVRA+M-CSF: Macrophages transfected with AV-BVRA virus and stimulated with M-CSF; AV+LPS: Macrophages transfected with AV-negative control virus and stimulated with LPS; AVBVRA+LPS: Macrophages transfected with AV-BVRA virus and stimulated with LPS.

As BVRA positive macrophages largely scattered in mice renal ischemia reperfusion injury, in order to mimic the renal injured microenvironment, we stimulated BVRA over-expressed macrophages with GM-CSF, M-CSF or LPS. GM-CSF and M-CSF were survival factors of macrophages. LPS was the major component Gram-negative bacteria cell wall. These factors are the major macrophage-related cytokines appearing in the renal microenvironment after injury. We found IL-10 expression was increased in BVRA over-expressed macrophages stimulated with GM-CSF (Figure 7A,D), M-CSF (Figure 7B,E) or LPS (Figure 7C,F); suggesting BVRA positive macrophage is a source of anti-inflammatory cytokine IL-10 in injured kidney.

## 3. Discussion

In this study, we first identified that BVRA expression was increased in M2 macrophage compared to M1 macrophage *in vitro*; BVRA positive macrophages were largely scattered in mice renal ischemia reperfusion injury, and BVRA over-expressed macrophages were associated with increased IL-10 expression; On the contrary, BVRA knockdown macrophages were associated with decreased IL-10 expression. BVRA over-expressed or knockdown macrophages had no effect on TNF-α expression, but BVRA over-expressed macrophages increased INOS and ARG-1 mRNA expression levels. This provides evidence of BVRA’s involvement in regulation of anti-inflammatory cytokines and its potential application in many diseases including inflammation, ischemia and kidney diseases.

Macrophages play a very important role in multiple functions, and can be polarized into pro-inflammatory (M1) (stimulated with GM-CSF) or anti-inflammatory (M2) (stimulated with M-CSF) by cytokines or environmental cues [18]. The M1 phenotype expressed the high production of reactive nitrogen and oxygen intermediates, promoting Th1 response. In contrast, M2 phenotype is characterized to be involved in parasite containment, promotion of tissue remodeling and tumor progression and to have immunoregulatory functions [19]. A differential cytokine production profile is a key feature of polarized macrophages. The M1 phenotype typically expressed a high level of IL-12 and a low level of IL-10, whereas M2 macrophages expressed more IL-10 but less IL-12 [20]. Such polarized processes are controlled by some key signaling molecules or genes, such as ARF [10], or TREM-1[11].

Biliverdin reductase (BVRA), an enzyme involved in the heme catabolic pathway, is also implicated in the oxidative stress response [21]. Apart from its antioxidative effects, a cytoprotective action independent of heme degradation has been reported [22]. Wegiel *et al.* reported that BV inhibited TLR4 expression and released of pro-inflammatory cytokines in macrophages, lack of BVRA result in increased TLR4 and TNF-α [15]. In kidney diseases, the injured renal tubular expressed and secreted the GM-CSF and M-CSF, directly fostering infiltration, proliferation, differentiation and survival of the bone marrow-derived macrophages in kidney, and was ultimately involved in kidney disease progression. In our study, we found that BVRA and IL-10 expression was increased in M2 phenotype macrophage compared to M1 phenotype macrophages. IL-10 expression was increased in BVRA over-expressed macrophages, while it decreased in BVRA knockdown macrophages. BVRA over-expressed or knockdown had no effect on TNF-α expression. However, BVRA over-expressed macrophages increased INOS and ARG-1 mRNA expression levels, thus we speculate that BVRA over-expressed macrophages were a group of particular macrophages. BVRA positive macrophages largely existed in kidney and bone marrow of mice renal ischemia reperfusion injury. We found GM-CSF, M-CSF or LPS, the macrophage-related cytokines from the renal injured microenvironment, promoted BVRA over-expressed macrophages to secrete anti-inflammatory cytokine IL-10, indicating BVRA in macrophages directly regulates the IL-10 expression.

Interleukin-10 (IL-10) has emerged as a key negative regulator of the immune system and is produced by a variety of immune cells, including macrophages, dendritic cells, T cells and B cells [23]. Typically, the effects of IL-10 are anti-inflammatory, such as its well-established ability to repress pro-inflammatory cytokine production by macrophages and dendritic cells. As a result, loss of IL-10 function results in increased severity of autoimmune or inflammatory diseases [24]. Previous study showed that in chronic kidney disease, IL-10 levels increase with worsening CKD stages [25]. IL-10 levels are elevated in patients with glomerular diseases, uremia [26], and hemodialysis [27]. Thus, BVRA may regulate the progression of chronic kidney disease by IL-10 pathway and may be a potential target of kidney diseases.

Other than a potent antioxidant responsible for the maintenance of intracellular redox homeostasis, BVRA was also identified as a serine/threonine/tyrosine kinase that modulates signal transduction pathways and regulates gene expression [12]. In previously study [14,21], BVRA was identified to protect against hypoxia by activation of PI3-Kinase and AKT. Kim *et al.* [22] reported that Tat-BLVRA protein was effective in inhibiting mitogen activated protein kinases (MAPKs), AKT and NF-κB activation. Wegiel *et al.* [28] found that cell surface biliberdin reductase mediates biliverdin-induced anti-inflammatory effects through enhanced production of IL-10. All these reports suggested that BVRA has the potential to alter the gene expression profile such as IL-10 in the macrophages.

In conclusion, our study evaluated the BVRA expression in macrophages polarization. We found that BVRA positive macrophages largely existed in renal ischemia reperfusion injury. BVRA regulates IL-10 expression in GM-CSF, M-CSF or LPS treated macrophages, indicating that BVRA is a potential target of kidney diseases via its effect on the IL-10 pathway in macrophages.

## 4. Experimental Section

### 4.1. Mice

Male C57BL/6 mice (aged 8–10 weeks) were purchased from Hua Fukang Company (Beijing, China). All animals were bred at Tongji Medical School, and all procedures were approved and performed in accordance with the institutional guidelines for animal care. 

### 4.2. Renal Ischemia Reperfusion Injury

Mice were anaesthetized with intraperitoneal injection of 1% sodium pentobarbital solution (5 mL/kg). Following back incision, the renal pedicle was bluntly dissected and a microvascular clamp was placed on the left renal pedicle for 30 min. Body temperature was controlled at 36.8–37.2 °C throughout the procedure.

### 4.3. Cell Culture

#### 4.3.1. L929 Cells 

The mouse fibroblast cell line L929 cells were purchased from the Cell Repository, Chinese Academy of Sciences. Cells were cultured in DMEM (Hyclone, Logan City, UT, USA) with 10% FBS (Hyclone) and incubated in a humidified atmosphere containing 5% CO_2_ at 37 °C.

#### 4.3.2. Preparation for Bone Marrow Derived Macrophages (BMDM)

BMDM were prepared from C57B/L6 mice as previously described. In general, bone marrow cells was isolated from femurs using aseptic technique and cultured for seven days in DMEM medium with 10% heat inactivated fetal bovine serum, penicillin (100 U/mL, KeyGEN, Nanjing, China), streptomycin (100 U/mL, KeyGEN) and 20% L929 cell-conditioned medium containing macrophage-colony-stimulating factors and other macrophages survival factors. Cells were further stimulated with GM-CSF (100 ng/mL, Peprotech, Rocky Hill, NJ, USA), M-CSF (100 ng/mL, Peprotech), LPS (100 ng/mL, Sigma-Aldrich, St. Louis, MO, USA), IFN-γ (100 ng/mL, Peprotech), IL-4 (10 ng/mL, Peprotech) or IL-13 (10 ng/mL, Peprotech) for 24 h and collected for further experiments.

### 4.4. Recombinant Adenoviruses Transfection

AV-BVRA was constructed, propagated and titred according to standard protocols by Shanghai Genechem Co., Ltd., using human 293A cells (ATCC, Rockville, MD, USA) expressing early region 1 (E1) adenoviral genes. AV-BVRA contains an expression cassette with the human cytomegalovirus (CMV) promoter, the mouse BVRA cDNA was fused with a GFP sequence in a polyA sequence. All adenoviruses amplification was by the Adeno-X™ Virus Purification Kit (BD Biosciences, Clontech, Mountain View, CA, USA) according to the manufacturer’s instruction. Viral titer was determined by plaque assay on 293A cells and expressed as PFUs. Transfection was performed in standard medium with 1% FCS for 48 h. Production of BVRA by transfected macrophages was assessed by Fluorescent counting method, Western blotting and qRT-PCR. AV-BVRA macrophages stimulated with GM-CSF (100 ng/mL, Peprotech), M-CSF (100 ng/mL, Peprotech) or LPS (100 ng/mL, Sigma-Aldrich) for 24 h.

### 4.5. Recombinant Lentivirus Transfection

The LV-BVRA vectors were constructed, amplified, and purified by Shanghai Genechem Co., Ltd. (Shanghai, China). The complete sequence of the mouse BVRA sequence was obtained from NCBI (GenBank Accession Number: NM_026678), a suitable shRNA target sequence was selected. The shRNA oligonucleotide fragments were annealed to form the target double-stranded DNA, insert the shRNA into the lentivirus expression vector. The plasmids were then transformed into competent *E. coli* DH5α cells and screened on plates containing antibiotics to produce the recombinant U6-vshRNA-UBI-GFP vector and the control lentivirus containing a non-silencing shRNA. The LV-BVRA vectors were packaged adopting Lenti-Easy Packaging Mix and Lentiviral Vector. On day one, a total of 6 × 10^6^ 293T cells were seeded in a 100 mm dish. On the following day, a transfection mix was made as the following: 25 µL Lenti-Easy Packaging Mix, 20 µL GFP Control Plasmid and 1455 µL Opti-MEM for a total of 1.5 mL for 5 min, 60 µL Lipofectamine, 2000 and 1440 µL Opti-MEM for a total of 1.5 mL for 5 min, and then were mixed together. This mix was added to the dish, and the cells maintained in 5% CO_2_ at 37 °C. All lentiviruses were concentrated and purified by Millpore Fast-trap Virus Purification and Concentation Kit (Millpore, Billerica, MA, USA). Viral titer was determined by plaque assay on 293T cells by Fluorescent counting method and qRT-PCR.BMDM transfection was performed in standard medium with 1% FCS for 24 h, then changed into standard medium with 10% FCS for 48 h. We determined their transfect efficiency by Western blotting and qRT-PCR.

### 4.6. Western Blotting

BMDM cells were lysed by RIPA lysis buffer containing cocktail protease inhibitors for 30 min on ice. Protein concentration was quantified by BCA assay kit (Pierce, Waltham, MA, USA). Equal amounts of protein (30 µg) was separated on 10% SDS-PAGE, and then transferred to nitrocellulose membranes. Membranes were blotted at 4 °C overnight with anti-BVRA antibodies (Stressgen, New York, NY, USA, 1:2000), anti-T–AKT (CST, Beverly, MA, USA, 1:1000), anti-P–AKT (CST, 1:1000), anti-T–ERK (CST, 1:1000), anti-P–ERK (CST, 1:1000), anti-T–JNK (CST, 1:1000), anti-P–JNK (CST, 1:1000), anti-INOS (Santa Cruz, CA, USA 1:250) and anti-ARG-1 (Santa Cruz, 1:250) followed by HRP-conjugated anti-rabbit IgG. The immunolabeled proteins were detected by enhanced chemiluminescense (Pierce). The density of bands was analyzed by ECL Assay Kit (Bipec Biopharma, Cambridge, MA, USA). Image J software was used to quantify the density of bands.

### 4.7. Quantitative RT-PCR

One microgram of cell total RNA was isolated by TRIZOL according to the manufacturer’s instructions (Invitrogen, Carlsbad, CA, USA), and was reverse transcripted into cDNA according to the manufacturer’s instructions (Promega, Madison, WI, USA). PCRs were performed on the Roche light 480II using the 2× SYBR master-mix (Qiagen, Dusseldorf, Germany). PCR thermal cycling parameters were: 95 °C for 10 min (PCR initial heat activation), and (two-step cycling) 95 °C for 10 s, 60 °C for 30 s (40 cycles). The analysis method was used ∆∆*C*_t_ methods. PCR primer sequences were presented in Table 1.

**Table 1:** Gene names and primer sequences used for qRT-PCR.

Gene Names	Primer Sequence
BVRA	Qiagen, Germany, QT00118762
ARG-1	Qiagen, Germany, QT00134288
IL-10	Qiagen, Germany, QT00106169
INOS	Qiagen, Germany, QT01547980
TNF-α	Qiagen, Germany, QT00104006
GAPDH	Qiagen, Germany, QT01658692
MMR(CD206)	Qiagen, Germany, QT00103012

### 4.8. Flow Cytometry

Cells were labeled for 30 min at 4 °С in FACS (Fluorescence Activated Cell Sorter) buffer (PBS contained 2% FBS and 0.02% azide) with manufacturer-recommended concentrations of purified rat anti-mouse CD45-APC-cy7 (Biolegend, San Diego, CA, USA), F4/80-PerCP-CY5.5 (Biolegend), CD11b-APC (ebioscience, San Diego, CA, USA) and anti-mouse BVRA-FITC (Jackson ImmunoResearch, West Grove, PA, USA) for 30 min. Cells incubated with FACS buffer alone were used as negative controls. The cells were analyzed by flow cytometry (FACSCalibur, BD Biosciences).

### 4.9. Elisa Assay 

Concentration of IL-10 and TNF-α in culture supernatants were quantified by ELISA assay kit (Biolegend) according to the manufacturer’s instructions. 

### 4.10. Immunofluorescence

Paraffin embedded mice IRI renal sections were blocked with 10% normal donkey serum for 1 h and then incubated with rabbit anti-mouse BVRA (stressgen, 1:50) and rat anti-mouse F4/80 (Abcam, Cambridge, UK, 1:50) overnight at 4 °C. After washing with PBS, the sections were incubated with donkey anti-rat Alexa Flour 488 and donkey anti-rabbit Cy3 (Jackson ImmunoResearch) conjugated secondary antibody, and washed with PBS. Sections were viewed and imaged with an Olympus fluorescence microscope and spot-cam digital camera (Diagnostic Instruments, Waltham, MA, USA).

### 4.11. Statistical Analysis

Statistical evaluation was performed by GraphPad Prism software, version 5.0. We used the nonparametric Mann-Whitney *U* test or unpaired *t*-test to evaluate *p* values. *p* values < 0.05 were defined as statistically significant.

## Supplementary Materials

Supplementary materials can be found at http://www.mdpi.com/1422-0067/16/09/22621/s1.
