# Supplementary material for: Biliverdin Reductase A (BVRA) Mediates Macrophage Expression of Interleukin-10 in Injured Kidney
Source: Int J Mol Sci. 2015 Sep 18;16(9):22621–35. doi: 10.3390/ijms160922621 (PMC4613327; doi:10.3390/ijms160922621)
Supplement: Supplementary file 1 [file ijms-16-22621-s001.pdf]

Supplementary Information

Macrophage polarization was induced by LPS and IFN-γ, IL-4 and IL-13, and BVRA expression increased in M2 macrophage, compared to M1 macrophage.

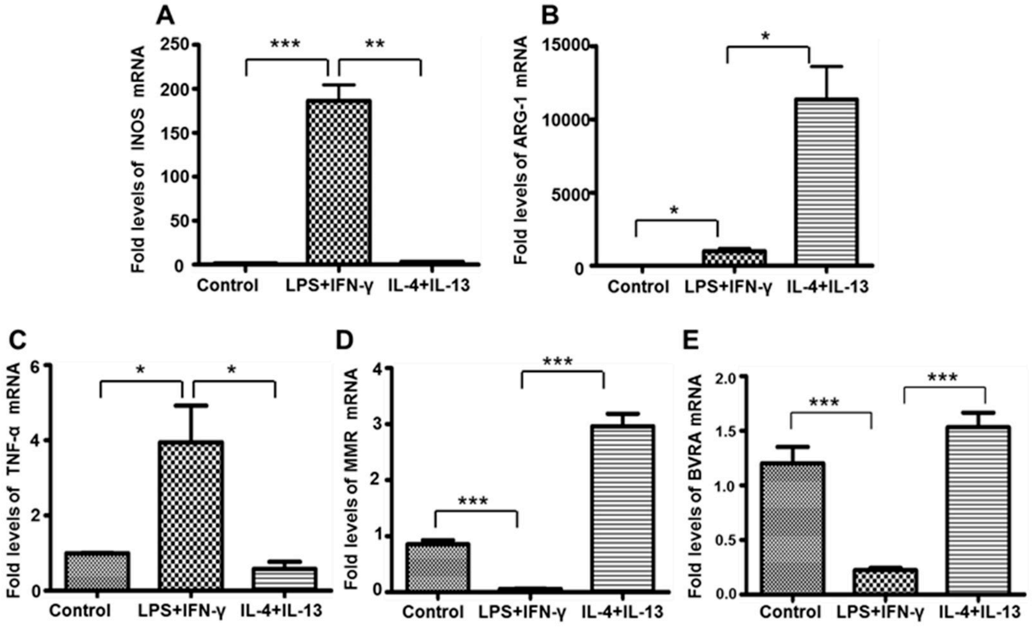

Figure S1. Macrophage polarization was induced by LPS and IFN-γ, IL-4 and IL-13. INOS (**A**); ARG-1 (**B**); TNF-α (**C**); MMR (**D**) and BVRA (**E**) mRNA expression was detected by qRT-PCR. Control: Macrophages with no treatment; LPS+IFN-γ: Macrophages with LPS 100 ng/mL and IFN-γ 100 ng/mL for 24 h; IL-4 + IL-13: Macrophages with IL-4 10 ng/mL and IL-13 10 ng/mL for 24 h. $n = 4$, *** $p < 0.001$, ** $p < 0.01$, * $p < 0.05$.

There was no difference between the AVBVRA group and control group in signal pathways' molecules such as P-AKT, T-AKT, P-ERK, T-ERK, P-JNK and T-JNK.

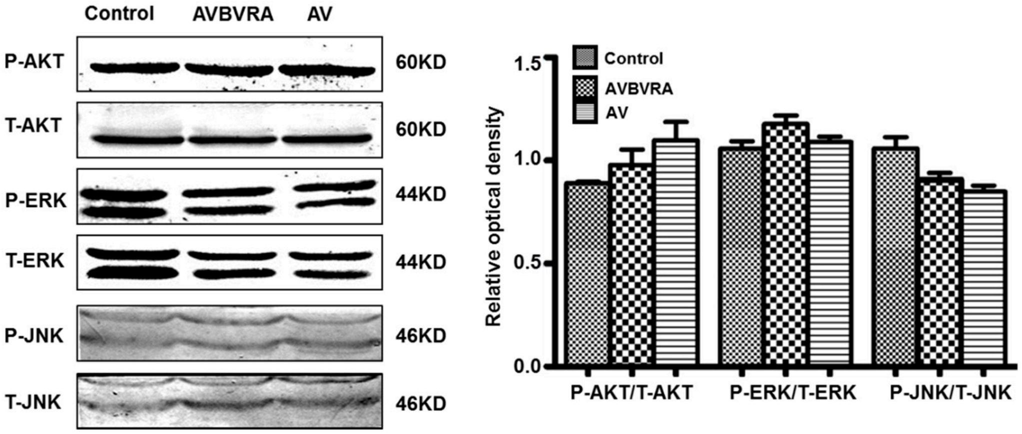

Figure S2. P-AKT, T-AKT, P-ERK, T-ERK, P-JNK and T-JNK protein were detected by Western blotting in over-expressed BVRA macrophages. Control: Macrophages with no virus; AVBVRA: Macrophages transfected with AV-BVRA; AV: Macrophages transfected with AV-negative control.
